# Supplementary figures and images for: Abundance of Poleroviruses within Tasmanian Pea Crops and Surrounding Weeds, and the Genetic Diversity of TuYV Isolates Found
Source: Viruses. 2022 Jul 30;14(8):1690. doi: 10.3390/v14081690 (PMC9416036; doi:10.3390/v14081690)

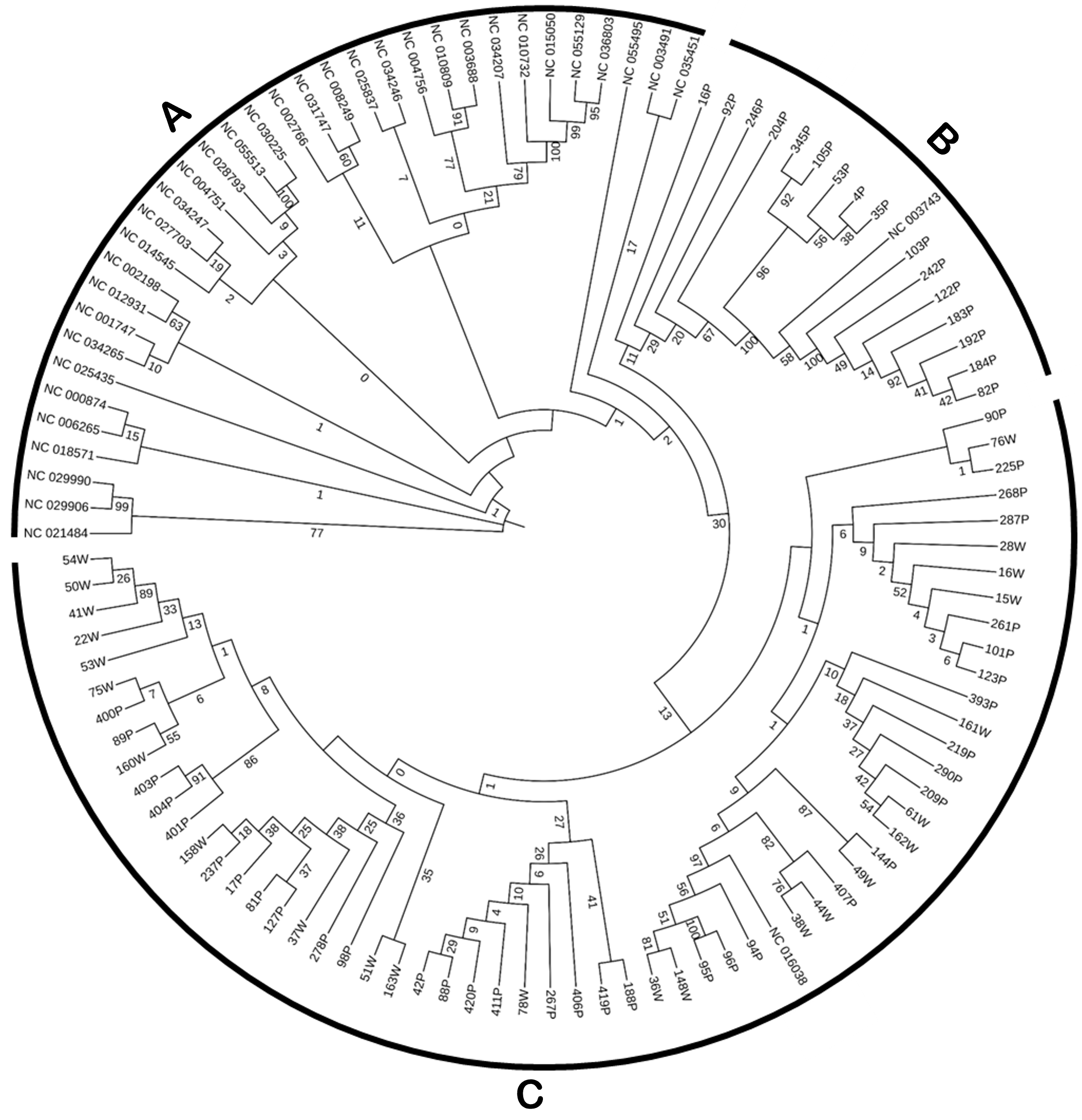

Supplement: Supplementary file 1 [file viruses-14-01690-s001.zip › Figure S1. P0 of Tasmanian TuYV isolates with P0 of all reported Poleroviruses.png]

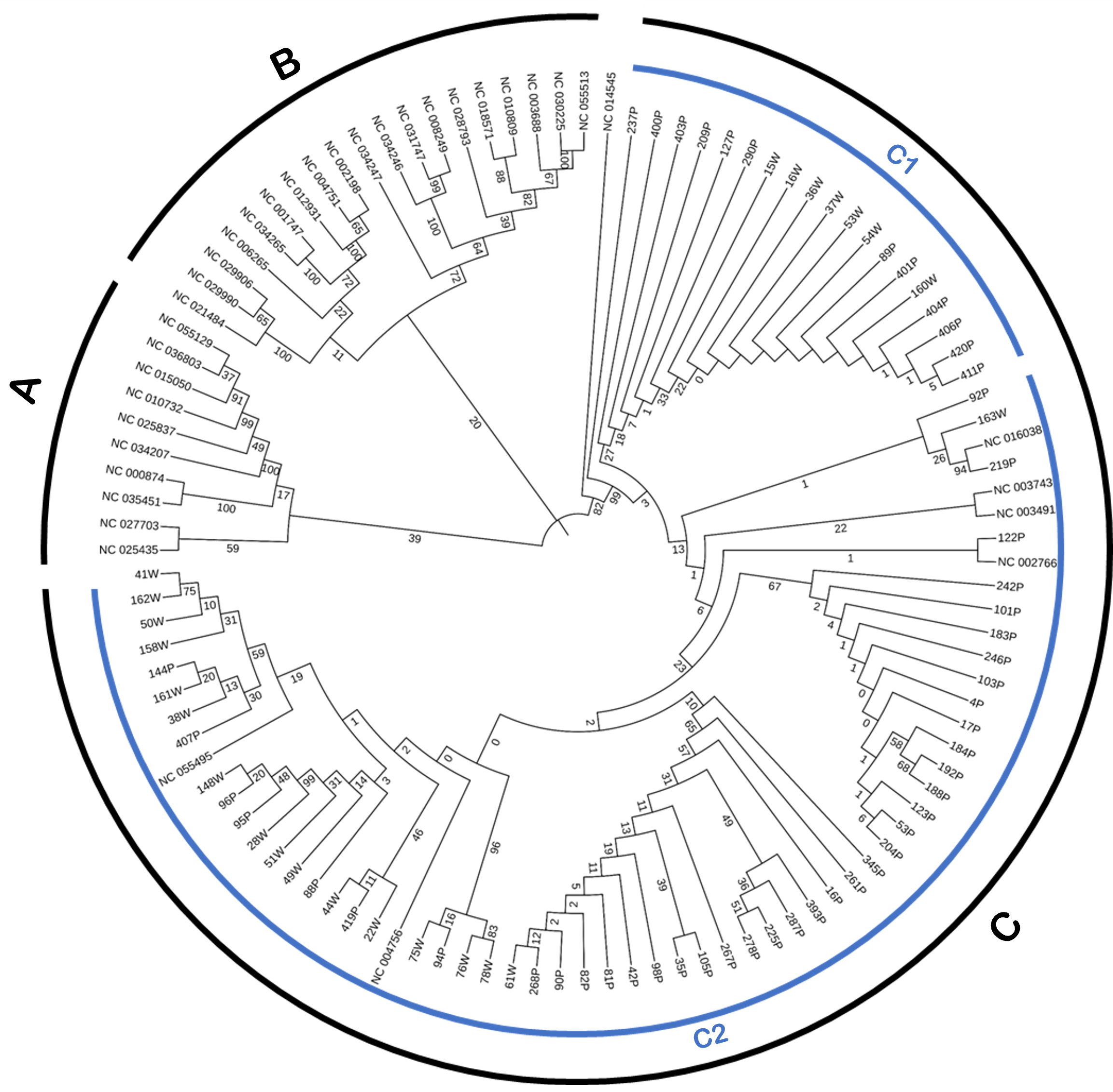

Supplement: Supplementary file 1 [file viruses-14-01690-s001.zip › Figure S2. CP of Tasmanian TuYV isolates with CP of all reported Poleroviruses.png]
